# Quantifying the Direct Transfer Costs of Common Brushtail Possum Dispersal using Least-Cost Modelling: A Combined Cost-Surface and Accumulated-Cost Dispersal Kernel Approach

**DOI:** 10.1371/journal.pone.0088293

**Published:** 2014-02-05

**Authors:** Thomas R. Etherington, George L. W. Perry, Phil E. Cowan, Mick N. Clout

**Affiliations:** 1 School of Environment, The University of Auckland, Auckland, New Zealand; 2 School of Biological Sciences, The University of Auckland, Auckland, New Zealand; 3 Landcare Research, Lincoln, New Zealand; The Australian National University, Australia

## Abstract

Dispersal costs need to be quantified from empirical data and incorporated into dispersal models to improve our understanding of the dispersal process. We are interested in quantifying how landscape features affect the immediately incurred direct costs associated with the transfer of an organism from one location to another. We propose that least-cost modelling is one method that can be used to quantify direct transfer costs. By representing the landscape as a cost-surface, which describes the costs associated with traversing different landscape features, least-cost modelling is often applied to measure connectivity between locations in accumulated-cost units that are a combination of both the distance travelled and the costs traversed. However, we take an additional step by defining an accumulated-cost dispersal kernel, which describes the probability of dispersal in accumulated-cost units. This novel combination of cost-surface and accumulated-cost dispersal kernel enables the transfer stage of dispersal to incorporate the effects of landscape features by modifying the direction of dispersal based on the cost-surface and the distance of dispersal based on the accumulated-cost dispersal kernel. We apply this approach to the common brushtail possum (*Trichosurus vulpecula*) within the North Island of New Zealand, demonstrating how commonly collected empirical dispersal data can be used to calibrate a cost-surface and associated accumulated-cost dispersal kernel. Our results indicate that considerable improvements could be made to the modelling of the transfer stage of possum dispersal by using a cost-surface and associated accumulated-cost dispersal kernel instead of a more traditional straight-line distance based dispersal kernel. We envisage a variety of ways in which the information from this novel combination of a cost-surface and accumulated-cost dispersal kernel could be gainfully incorporated into existing dispersal models. This would enable more realistic modelling of the direct transfer costs associated with the dispersal process, without requiring existing dispersal models to be abandoned.

## Introduction

Dispersal is an important process for ecology and evolution, affecting organisms at the individual, population, and species levels by influencing population dynamics and gene flow. Conceptually, dispersal can be viewed as a combination of three stages: departure, transfer, and settlement. Each stage imposes energy, time, risk, and opportunity-based costs on the disperser that can either be direct and incurred immediately, or indirect and incurred subsequently [1]. These costs need to be quantified from empirical data and incorporated into dispersal models to improve our understanding of the dispersal process [2].

**Figure 1:**
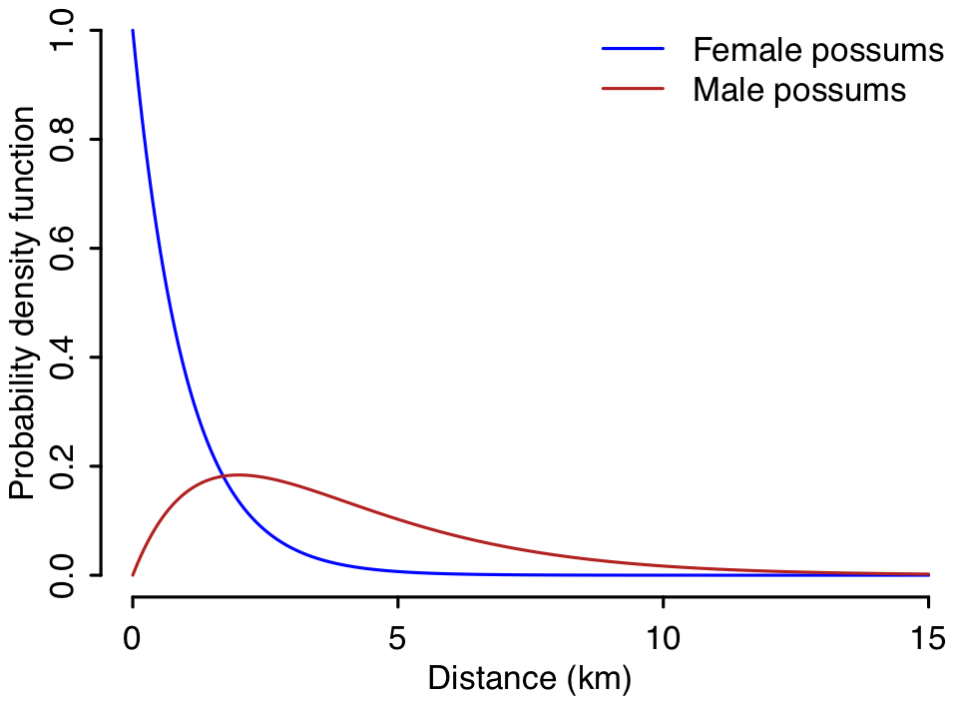
Straight-line distance based dispersal kernels. These dispersal kernels are used to model dispersal for male and female brushtail possums (*Trichosurus vulpecula*) in New Zealand [52].

We are interested in quantifying the direct costs associated with the transfer stage of dispersal, in which an organism moves from one location to another. Traditionally a distance-based dispersal kernel, calibrated from data on straight-line distances between the start and end locations of known dispersal events (Figure 1), is used within dispersal models to quantify the likelihood of an organism dispersing a given distance in any direction [3]. However, this approach assumes that the landscape does not affect dispersal directions or distances. Wiens [3] presents a contrasting view, where during the transfer stage of dispersal “a landscape can be viewed … as a cost-benefit surface in which there are ‘peaks’ where benefits outweigh costs and ‘valleys’ where costs exceed benefits”. This representation of the landscape as a cost-benefit surface fits nicely with both the conceptual model of the direct energy, time, or risk costs associated with the transfer stage of dispersal [1], and also with measuring connectivity using least-cost modelling [4], [5].

Least-cost modelling is based upon a geographic information system (GIS) raster called a cost-surface (otherwise known by combinations of: cost, friction, permeability, or resistance, and; layer, grid, map, raster, or surface). Cost-surfaces are developed using a variety of approaches [6] and are used to represent the difficulty associated with traversing different parts of a landscape. Cells with higher costs represent species-specific factors such as greater mortality risk, energy expenditure, or behavioural aversion that impede movement. Given a starting cell location, least-cost modelling calculates least-cost paths (LCPs) that are routes of maximum efficiency from the start cell to each other cell as a function of the distance travelled and the costs traversed. Connectivity between the start and end cell are expressed using the accumulated-cost of the LCP.

Given a cost-surface and the same start and end locations of known dispersal events that are typically used to calibrate a distance based dispersal kernel, we can envisage a least-cost modelling approach to quantifying direct transfer dispersal costs. If LCPs are calculated between the start and end locations, then the accumulated-cost of the LCPs could be used to calibrate an accumulated-cost based dispersal kernel. This novel combination of a cost-surface and its associated accumulated-cost dispersal kernel would then enable the transfer stage of dispersal to incorporate the effects of landscape features, by modifying the direction of dispersal based on the cost-surface and the distance of dispersal based on the accumulated-cost dispersal kernel.

Therefore, in contrast to the straight-line distance approach to modelling dispersal that assumes that the landscape does not affect dispersal directions or distances, a least-cost modelling approach to quantifying the direct transfer costs of dispersal would enable landscape features to be taken into account. This would help in moving towards a new generation of dispersal models that are more empirically based [2]. Such a change would not even require existing dispersal models to be abandoned, as it would only be the way in which an organism disperses from a start to an end location that changes, meaning existing dispersal models could be easily adapted.

**Figure 2:**
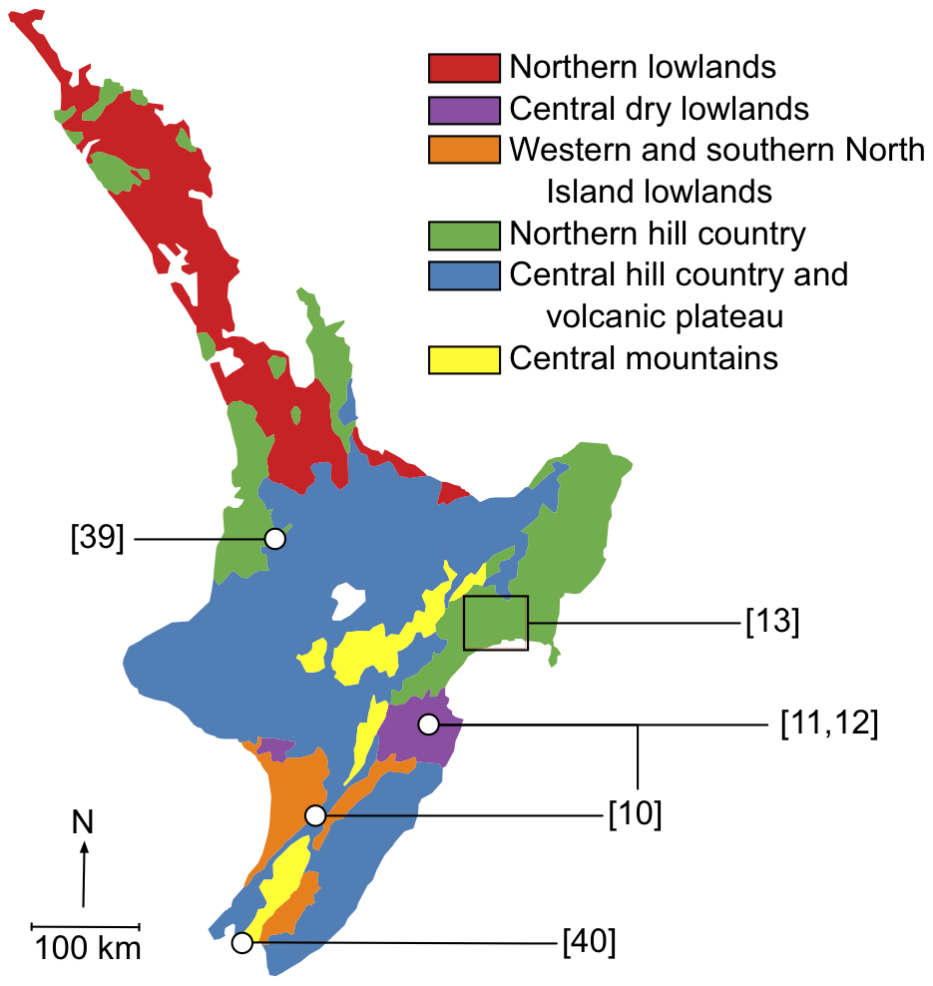
The locations of six studies that provided empirical possum dispersal data. Dispersal of possums was measured either directly using radio-telemetry or indirectly using landscape genetics, and each study was associated with a variety of different landscape environments across the North Island of New Zealand categorised on the basis of differences in climate, landform, and soils [53].

To demonstrate how the direct transfer costs of dispersal can be quantified using least-cost modelling, we apply our proposed approach to the common brushtail possum (*Trichosurus vulpecula*), a nocturnal marsupial, about 70–90 cm in length and 2–3.5 kg in weight, which has become a notorious invasive species since its introduction to New Zealand from Australia during the late 1800s and early 1900s [7]. Using empirical dispersal data collected by six dispersal studies located across the North Island of New Zealand (Figure 2), we demonstrate how it is possible to develop a cost-surface and an associated accumulated-cost dispersal kernel.

## Methods

Our least-cost modelling approach consisted of three main steps. First, a set of GIS rasters that represented landscape features likely to affect possum dispersal were created. Second, a widely used landscape genetics approach that compares the genetic distance and LCP accumulated-cost between locations was used to calibrate, rank, and evaluate a variety of cost-surfaces made from differing combinations of landscape features and costs. Finally, in the novel third step of our approach, accumulated-cost dispersal kernels for the best cost-surfaces were calibrated based on the LCP accumulated-cost between the start and end locations of dispersal events recorded using radio-telemetry.

### GIS data processing

Based on a review of possum movement and dispersal studies [8]–[13], we began by creating six GIS raster datasets that represented landscape features that we thought were likely to affect possum dispersal (Table 1). The same GIS data processing was applied to produce sets of rasters that represented the landscape features of interest across each of the possum dispersal data study areas (Figure 2). All source GIS data were at a map scale of 1∶50,000 and in all cases the data used were as contemporary as possible to the time of collection of the possum dispersal data from each study. Choice of scale, in terms of both grain and extent, is important for any spatial analysis. We chose extents for each possum dispersal study that were as small as possible to reduce computation times, but which provided sufficient space around data points to avoid any edge effects that might influence the least-cost modelling. For grain, as possums are strictly nocturnal we considered any dispersal event to be the sum of a series of nightly movements. Therefore, we set the cell resolution of all the rasters at 70.5 m, which produced cells with an area equal to the minimum observed nightly range of possums [14].

**Table 1:** The six GIS raster datasets representing the landscape features thought to affect possum dispersal, the ecological justification for their choice, and the associated weight ranges that represent the perceived relative importance of each landscape feature.

GIS dataset	Dispersal affect	Weight range
Elevation	Aversion to higher colder elevations.	0.1–10
Plan curvature	Preference for drier ridges over wetter gullies.	0.1–10
Tree and scrub cover	The absence of cover provided by tree and scrub appears to modify movement behaviour.	0.1–10
Highway traffic volume	Highways with higher traffic volumes present an obstacle to dispersal that acts through behavioural aversion and direct mortality.	10–100
River order	While possums can swim, and have dispersed across rivers, they are disinclined to enter water, so high order river are thought to be a serious obstacle dispersal.	100–1000
Bridge length	Bridges enable possums to avoid the cost associated with crossing a river, but they may also increase the risk of mortality associated with highways as a function of length.	0.1–100

The topographic landscape features of elevation and ridges were both derived from a digital elevation model (DEM) [15]. To identify ridges we calculated continuous plan curvature values for which greater positive values represent more pronounced ridges and greater negative values represent more pronounced channels [16]. A cubic spline interpolation was used to resample the elevation and plan curvature values to the desired resolution.

Tree and scrub cover for each area where possum dispersal data were gathered was rasterised from the most contemporary of New Zealand's landcover databases [17] to produce a raster in which cell values represented the percentage of the cell consisting of tree and scrub landcover.

Rivers were differentiated by stream order, assuming that stream order is relative to river characteristics such as channel size and discharge [18]. Less ephemeral main rivers with a stream order greater than four were extracted from a DEM-derived river drainage dataset [19]. The spatial accuracy of the drainage dataset was poor at the desired map scale, therefore the river order values were transferred to the matching river and canal centreline and polygon objects within the watercourse entity class of the national topographic database [20], [21]. Once rasterised, an algorithm [22] was run to ensure that no gaps in the river order values had been introduced during data processing. All rail and road bridges from the bridge entity class of national topographic database [20], [21] that were associated with any of the main rivers were also rasterised, with the cells attributed with the length of the bridge's vector geometry.

Highway traffic volumes were based on point estimates of annual average daily traffic (AADT) for state [23] and regional highways [24], [25]. The AADT values were attributed to an appropriate vertex within a highway network that, because of temporal changes to the highway network, was developed where appropriate from a combination of: road centreline objects within the road entity class of the national topographic database [20], [21], state highway road data [26], and manual digitisation. The remaining highway network vertices received an AADT value by using a network-based spatial interpolation approach [27], in which the interpolated values were calculated from an inverse distance weighted interpolation [28] based on the distance along the highway network to the first set of AADT values that were encountered. The highway network's AADT values were then rasterised.

All offshore areas and any features from the lagoon and lake entity classes of the national topographic database [20], [21] that were at least the area of a single 70.5 m cell were rasterised, and these areas were treated as null data in the cost-surface, to represent complete barriers to possum dispersal.

**Figure 3:**
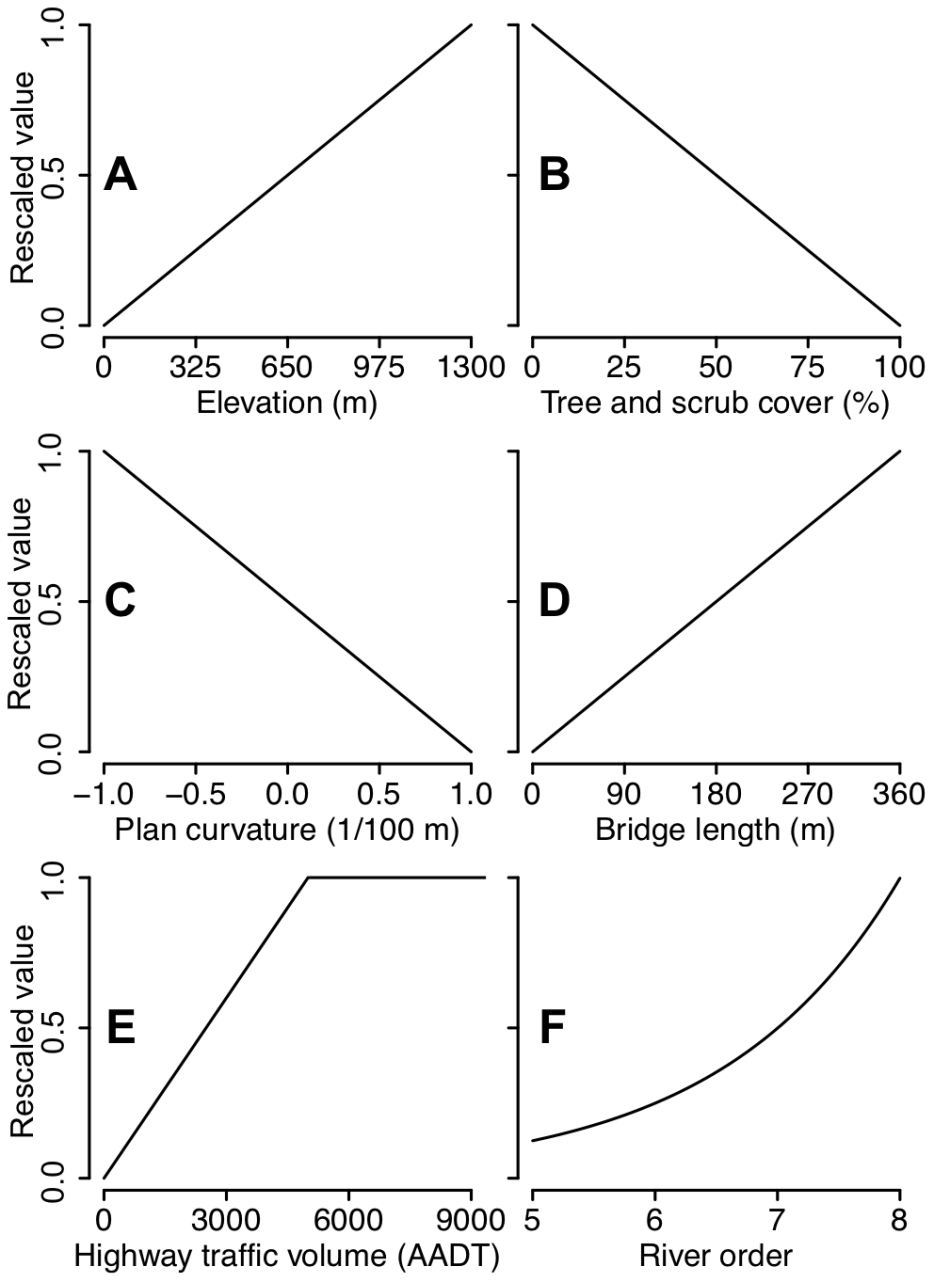
Intra-feature cost-surface weightings. The form of the relationships used to rescale the values of each landscape feature to between zero and one, and to control the intra-feature weighting of the values from each landscape feature.

So that the set of rasters could be combined into a cost-surface, each raster's values were rescaled to between zero and one such that a set of relative inter-feature weights could be applied. This rescaling also provided an opportunity to control the intra-feature weighting of each landscape variable. With limited knowledge regarding how possum dispersal may vary across the range of values associated with each of the features, we assumed a linear function for rescaling the values of elevation, plan curvature, tree and scrub cover, and bridge length between zero and one (Figure 3 A–D).

Given evidence from of the barrier effect from traffic volumes [9], we assumed a linear increase from zero to 5000, and that all values from 5000 upwards were equal to one (Figure 3E). As the formation of a river of order *n* requires two rivers of order *n*-1 [18], we used a function in which a river of order *n* was half that of a river of order *n*+1 (Figure 3F). Also, on occasions when rivers and bridges were included in the same cost-surface, any river cells that coincided with bridge cells were reclassified to zero to remove the effect of rivers at these locations.

While other studies have varied the intra-feature weightings [29], we chose not to do this as it would have resulted in a factorial increase in the number of landscape representations, and we did not feel we had any sensible alternatives to the linear functions used where the relationship was not clear.

### Cost-surface calibration and ranking

We used a commonly applied two-stage expert opinion-landscape genetics approach [6] to calibrate a set of potential cost-surface landscape representations. The possible range of relative inter-feature weights (Table 1) was set based on our own interpretation of the same studies that informed the choice of landscape features affecting possum dispersal [8]–[13]. Due to the uncertainty associated with this process we limited ourselves to prescribing ranges of possible weights using orders of magnitude (0.1, 1, 10, 100, 1000) that were constrained only by our perceived rank importance of the variable, as this is important for robust least-cost modelling [30].

We then defined 47 different cost-surface landscape representations that were logical combinations of the six landscape features. A landscape genetics based optimisation approach in which multiple cost-surfaces are compared statistically to identify the cost-surface that best explains the genetic distances [31], was used to select an appropriate set of inter-feature weights for each cost-surface landscape representation. We used data from a landscape genetics possum dispersal study [13] that had measured genetic distances, in the form of linearised *F*_ST_ [32], between possums sampled at 31 different locations.

The landscape genetics based optimisation approach is computationally very demanding, which limits the optimisation process [6]. Therefore, to reduce computation times instead of using a more conventional matrix approach, which would require that for each cost-surface LCP accumulated-costs would need to be calculated between all 465 possible combinations of possum sampling locations, we restricted our analysis to a network of neighbouring possum sampling locations. We defined neighbours using a Delaunay triangulation [28], but with neighbours that were greater than 25 km apart removed, as the genetic signal disappears beyond this point [13]. This created a sample of 77 neighbouring possum sampling locations between which genetic distance and LCP accumulated-cost from different cost-surfaces could be compared. Limiting the landscape genetics analysis to a network of neighbouring locations not only reduces computation times, but may also improve the ability to detect a meaningful pattern between the landscape and the genetics [33], and enables for robust statistical techniques such as regressions and Akaike's Information Criterion (AIC) to be used to assess different cost-surface landscape representations [34].

To find a best fitting set of weights for each of these cost-surfaces, we generated a Latin hypercube sample (LHS) [35] of size 1000 for the weight ranges, as this provides an efficient way of sampling a multi-dimensional parameter space to look for main effects. Each of the 1000 LHS weight samples were applied to each of the 47 cost-surface landscape representations using a map algebra point operation (Equation 1) to create a cost-surface (*cs*) from the set of raster landscape features that made up a cost-surface (*S*) by multiplying each raster (*r*) by its associated weight (*w_r_*). 
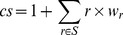
(1)


To determine the best fitting set of inter-feature weights for each of the 47 cost-surfaces, the computational efficient irregular landscape graph approach [36] was used to calculate LCP accumulated-cost between possum sampling locations. The ecological grain of the irregular landscape graphs was based upon habitat specific measures of home-range size [14], the very important point threshold was set at one, and the possum sampling locations were included as points of interest. The best fitting set of weights from the 1000 LHS weight samples for each of the 47 possible cost-surface landscape representations was identified as that which produced the smallest residual sum of squares (RSS) from a linear regression between genetic distance and LCP accumulated-cost. As the sets of weights being compared had produced accumulated-costs with differing orders of magnitude, the accumulated-costs were first rescaled to range between zero and one to enable a fair comparison.

The 47 best fitting cost-surface landscape representations were then ranked by the corrected small sample AIC (AICc) that was calculated directly from the RSS values [37]. We also calculated the Akaike weight (*w*_i_) to better interpret the relative strength of evidence for each landscape representation [37], and the coefficient of determination (*r* ^2^) from the linear regressions to assess the explanatory power. As well as ranking the 47 cost-surfaces landscape representations, we also included a uniform landscape representation in which connectivity between the possum sampling locations was measured using straight-line distance rather than LCP accumulated-cost. As with the cost-surfaces, an RSS value was also calculated by fitting a linear regression between the rescaled distances and the genetic distances.

To ensure the *w*_i_ and *r* ^2^ values derived from RSS value of each linear regression were reliable, each set of linear regression residuals were examined for signs of spatial autocorrelation. As the residuals were associated with links within a network of neighbouring possum sampling locations, the spatial structure between the residuals was defined as a binary variable representing adjacency of the links within the network. The level and significance of spatial autocorrelation was measured using a randomisation based global Moran's I [38].

### Dispersal kernel calibration and comparison

A dispersal kernel was calibrated for each of the landscape representations using the start and end points of dispersal events from 65 (47 ♂ and 18 ♀) radio-collared possums across five different studies [10]–[12], [39], [40]. A dispersal event was defined as a movement greater than 2 km, as given the size and shape of possum home-ranges movements shorter than this could have represented within home-range movements rather than the dispersal transfer movements between home-ranges that we were trying to quantify [41].

Dispersal connectivity was calculated between the start and end points of all of the radio-collared possum dispersal events as either straight-line distance for the uniform landscape representation, or as LCPs for the 47 cost-surface landscape representations. Mann-Whitney *U*-tests were used to check for differences in dispersal connectivity values between sexes, in order to assess whether different kernels were required for each sex. The dispersal kernel for each landscape representation was defined by a lognormal probability distribution fitted to the dispersal connectivity values of the possum dispersal events via maximum-likelihood [42].

To compare the differing quantifications of dispersal transfer costs associated with the traditional straight-line distance approach and our proposed least-cost modelling approach, we produced maps that visualised the likely dispersal directions and distances given an arbitrary starting location.

## Results

**Figure 4:**
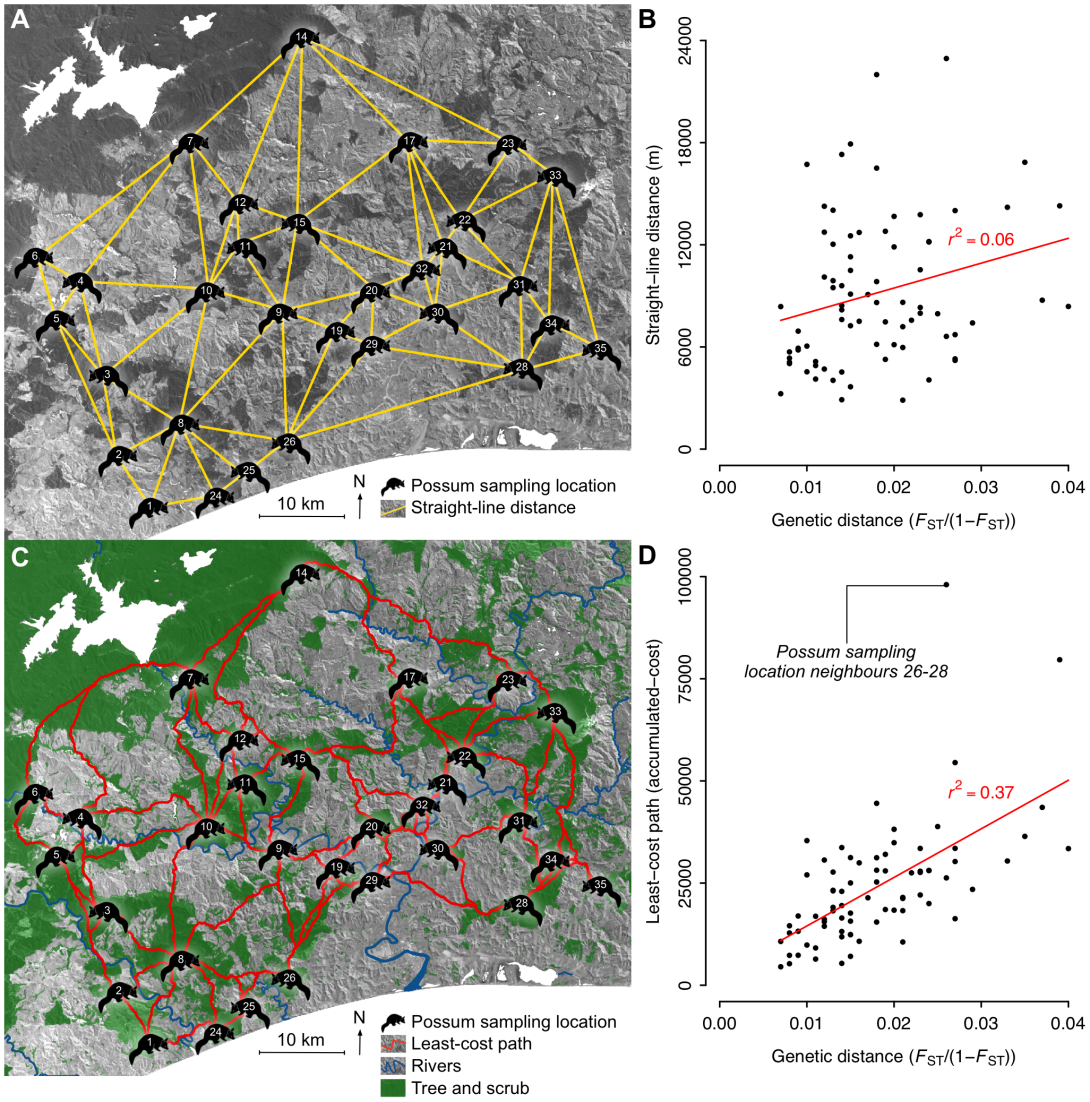
Comparing genetic distance with straight-line distance and accumulated-cost. Examples of two of the 48 landscape representations used to measure the direct transfer costs between 77 neighbouring possum sampling locations for which genetic distances had been measured. Assuming (A) a uniform landscape representation (D, in Table 2), connectivity was measured as straight-line distance that (B) explained little of the variation in genetic distances. By using (C) a cost-surface landscape representation that incorporated transfer costs associated with rivers and the absence of tree and scrub cover (DTR, in Table 2), connectivity was measured as the accumulated-cost of least-cost paths that (D) explained approximately a third of the variation in genetic distance.

The landscape genetics-based AIC ranking of the landscape representations (Table 2) showed little support for the uniform landscape representation (D, in Table 2) for which connectivity was measured using straight-line distance (Figure 4A). This representation ranked 46 out of 48, with the *w*_i_ and *r* ^2^ values indicating that there was minimal evidence to support this option as it does not explain differences in genetic distance (Figure 4B). This was in stark contrast to the highest ranked cost-surface landscape representation (DTR, in Table 2) for which connectivity was measured using the accumulated-cost of LCPs (Figure 4C). The *w*_i_ and *r* ^2^ values of this representation indicated that there was strong evidence to support this option, as over a third of the variation in genetic distance could be explained by LCP accumulated-cost (Figure 4D).

**Table 2:** A summary of the landscape genetics based ranking of the 48 landscape representations, with the parameterisation of the associated accumulated-cost dispersal kernel.

Representation a	E	P	T	R	H	B	Rank b	*w*_i_	*r* ^2^	μ_log_	σ_log_	maxDC
DTR	-	-	4.78	445.43	-	-	1	0.42	0.37	9.74	0.71	79863
DR	-	-	-	259.29	-	-	2	0.14	0.31	8.53	0.59	24492
DTRH	-	-	4.14	345.80	24.40	-	3	0.12	0.34	9.69	0.69	73144
DPTR	-	2.65	4.67	793.76	-	-	4	0.07	0.34	10.08	0.66	114941
DRH	-	-	-	259.82	13.33	-	5	0.07	0.31	8.57	0.57	25564
DPR	-	5.22	-	981.20	-	-	6	0.05	0.32	9.73	0.63	89911
DPTRH	-	2.44	6.37	638.90	97.94	-	7	0.03	0.32	10.33	0.67	134542
DER	0.11	-	-	319.56	-	-	8	0.02	0.32	8.55	0.60	27353
DPRH	-	4.73	-	830.30	33.00	-	9	0.01	0.31	9.71	0.61	82445
DETR	0.19	-	3.88	476.05	-	-	10	0.01	0.34	9.58	0.72	72338
DEPR	0.15	4.88	-	950.56	-	-	11	0.01	0.32	9.68	0.63	86879
…												
D	-	-	-	-	-	-	46	0.00	0.06	8.33	0.52	12799

a Each landscape representation measured connectivity as a function of distance (D) and the traversal costs resulting from the inter-feature weights of elevation (E), plan curvature (P), tree and scrub cover (T), river order (R), highway traffic volume (H), and bridge length (B).

b Landscape representations were ranked by their Akaike weight (*w*_i_). Only the top ranked cost-surface landscape representations (Σ*w*_i_≤0.95) plus the uniform landscape representation are listed.

*r* ^2^  =  coefficient of determination (in all cases *p*≤0.03), μ_log_  =  the mean of the logarithm of the lognormal dispersal kernel, σ_log_  =  the standard deviation of the logarithm of the lognormal dispersal kernel, maxDC  =  maximum observed dispersal connectivity value.

Analyses of the spatial autocorrelation of the regression residuals support these results. There was only slightly positive and non-significant spatial autocorrelation present for the top ranked cost-surface landscape representations (in all cases Moran's I = 0.05–0.07, *p*≥0.05 one-tailed) indicating that the *w*_i_ and *r* ^2^ values can be interpreted with confidence. In contrast the uniform landscape representation's residuals had significant positive autocorrelation (Moran's I = 0.20, *p*<0.01 one-tailed), indicating that some spatial factor that influences genetic distances is not accounted for – which we would argue is clearly the effect of landscape features.

We felt confident that the range of values explored for the inter-feature weights were appropriate as the weights selected for the top ranking landscape representations were well within the range of values explored, suggesting that a wider range of values may not have produced different results. Based on their occurrence in the top ranking landscape representations and the *w*_i_ values, the most important landscape feature was river order, followed by tree and scrub cover, with some support for highway traffic volume.

**Figure 5:**
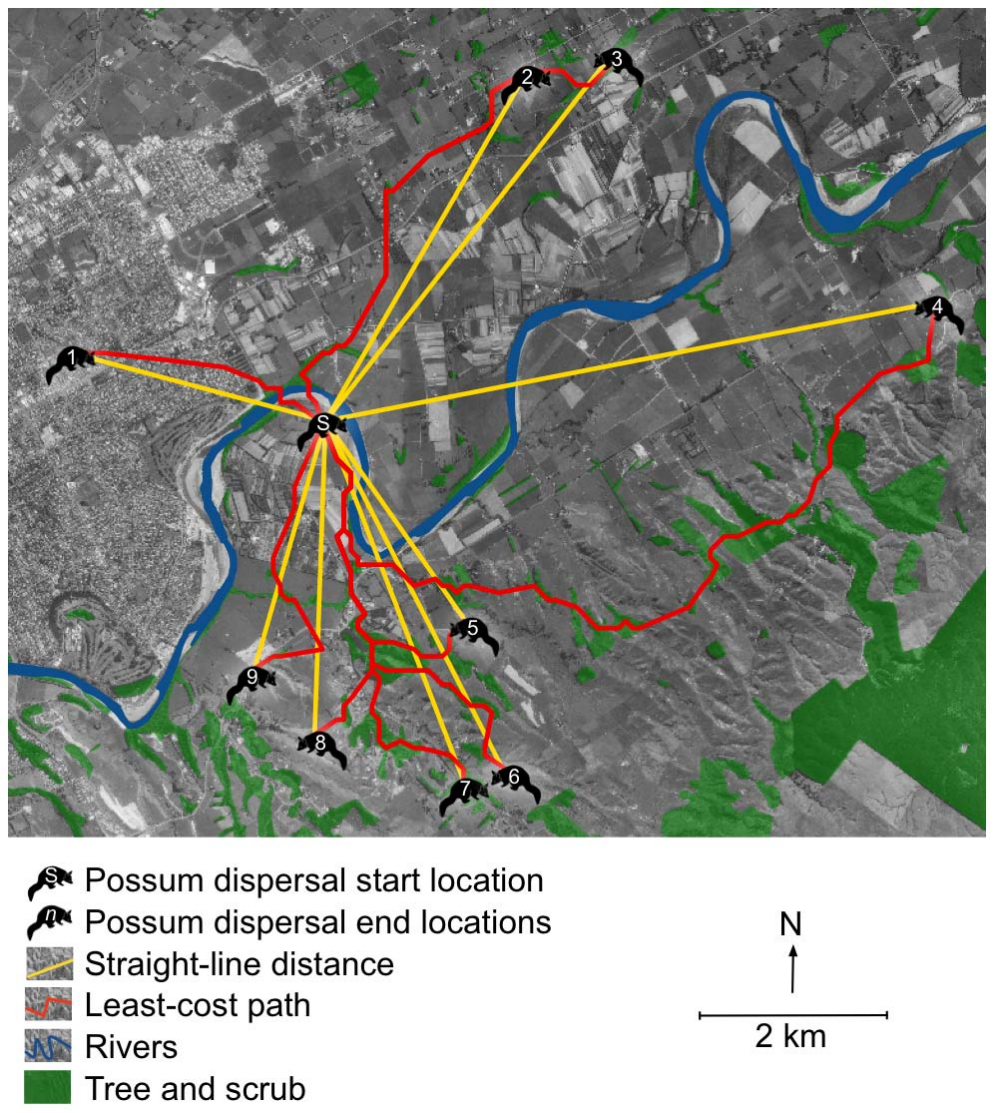
Dispersal events as straight-lines and least-cost paths. Examples of nine of the 65 radio-collared brushtail possum dispersal events used to calibrate the dispersal kernels. To allow for the effect of incorporating landscape features to be shown, both the straight-line distances associated with the uniform landscape representation (D, in Table 2) and the least-cost paths associated with the highest ranked cost-surface landscape representation (DTR, in Table 2) are shown. While the straight-line distances do not consider the landscape at all, the least-cost paths avoid both rivers and areas without tree and scrub cover.

**Figure 6:**
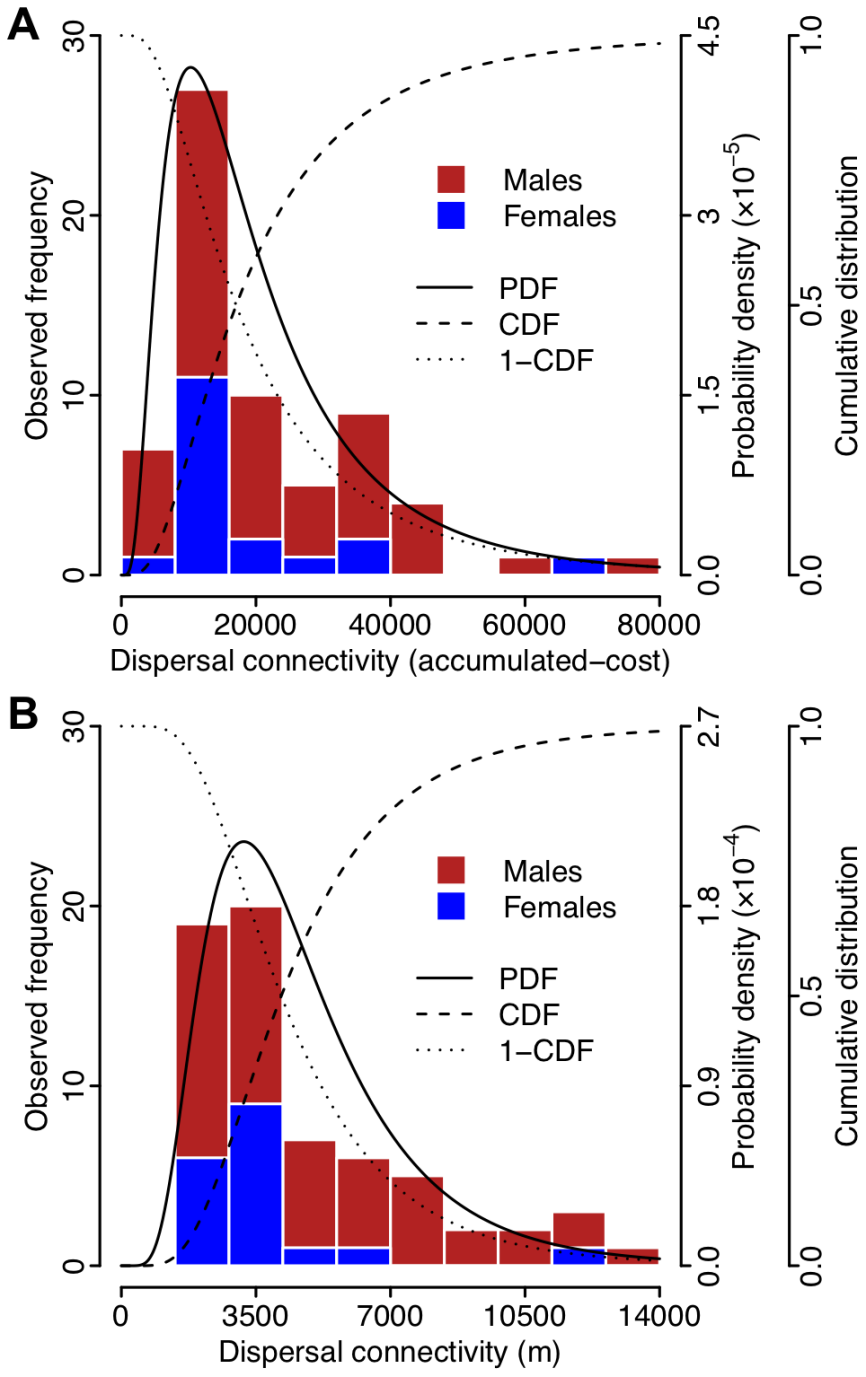
Possum dispersal kernels based on straight-line distance and accumulated-cost. Examples of the dispersal kernel calibration from connectivity values of observed possum dispersal events for (A) the highest-ranked cost-surface landscape representation (DTR in Table 2), and (B) the uniform landscape representation (D in Table 2). A stacked histogram of the possum dispersal events categorised by sex is shown along with the fitted lognormal dispersal kernel's probability density function (PDF), and both the cumulative distribution function (CDF) and the inverted cumulative distribution function (1-CDF).

There were notable differences for dispersal events when measured using straight-line distance for the uniform landscape representation, or LCPs for the cost-surface landscape representations (Figure 5). The dispersal connectivity values did not tend to differ between sexes for any of the landscape representations (in all cases Mann-Whitney *U*≥345, *p*≥0.26 two-tailed). Therefore, lognormal dispersal kernels were fitted to the dispersal connectivity values derived for each landscape representation with the two sexes combined (Table 2, Figure 6).

**Figure 7:**
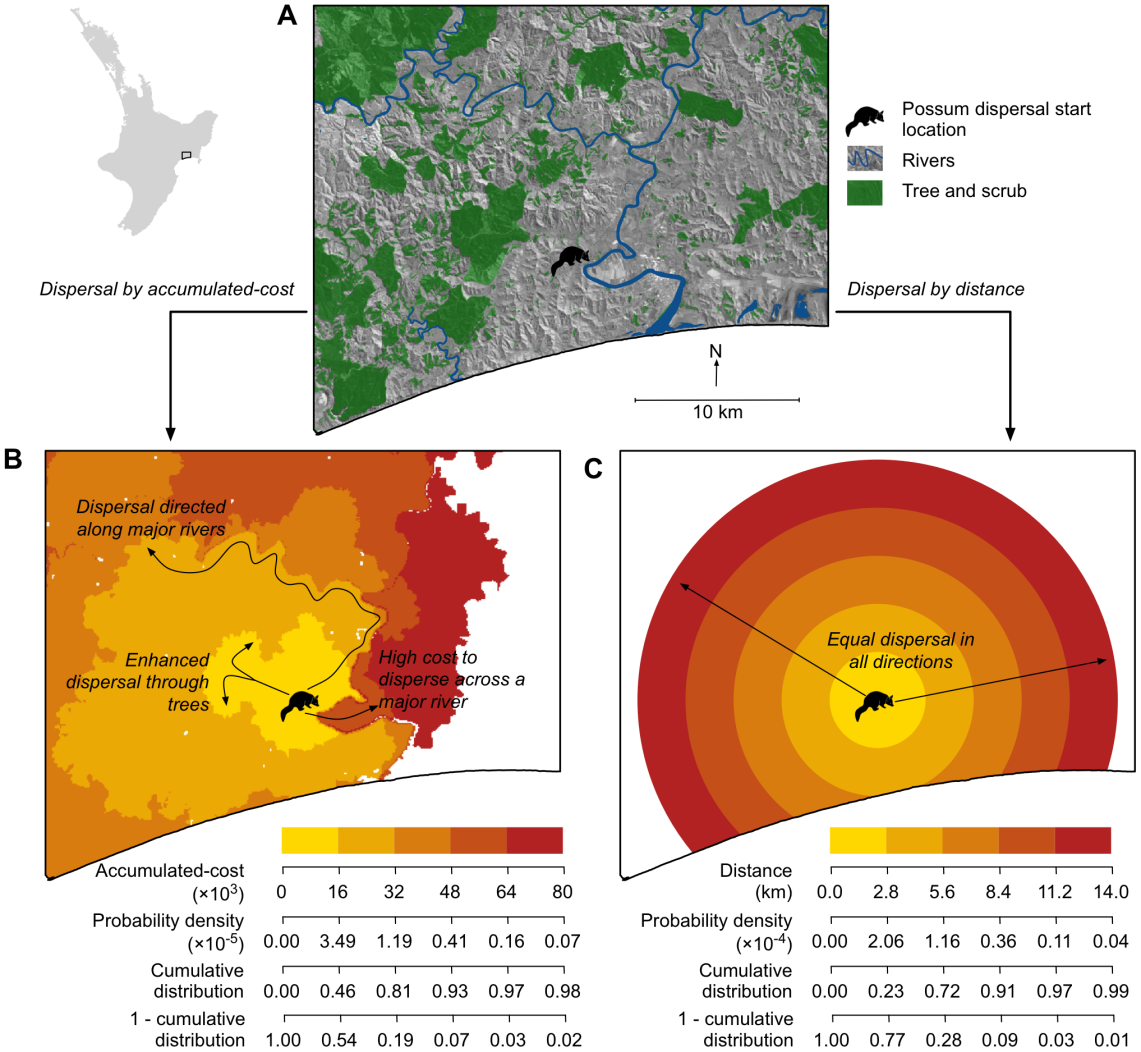
Examples of least-cost modelling versus straight-line distance based dispersal models. For (A) an arbitrary section of landscape and possum dispersal starting location, dispersal is modelled using (B) the highest-ranked cost-surface landscape representation (DTR in Table 2) for which connectivity is measured by accumulated-cost, and (C) the uniform landscape representation (D in Table 2) for which connectivity is measured by distance. The connectivity values converted to the associated probability density and cumulative distribution values given the associated dispersal kernel (Figure 6).

The fact that the least-cost modelling approach to quantifying direct transfer costs was able to incorporate landscape features into the likely dispersal directions and distances was evident when these costs were visualised (Figure 7B). This ability was in stark contrast to the straight-line distance approach in which dispersal trajectories are clearly independent of important landscape features (Figure 7C).

## Discussion

The LCP accumulated-cost connectivity values derived from our cost-surface landscape representations explained about one-third of the variation in genetic differentiation (Table 2). These outcomes were heartening given the general limitations with using a landscape genetics approach to quantify connectivity across a landscape [31], to which we could add more specific issues such as geographical variability in possum control operations which could not be accounted for. However, we do not preclude the possibility that there could be cost-surfaces that are better representations of the landscape. For example, the extreme outlying data point for possum sampling location neighbours 26–28 that was present for the best landscape representation (Figure 4D) would suggest that the landscape representation could possibly be improved. Such difficulties in cost-surface calibration are not uncommon, which emphasises the importance of considering uncertainty when using cost-surfaces in any ecological application [6].

Assuming that our cost-surfaces are a reasonable representation of the actual traversal costs for possums on the North Island of New Zealand, our results are conclusive in demonstrating that a uniform landscape representation in which dispersal can be based upon straight-line distance movement is far too simplistic. We feel confident that this will be the case for other species and locations as previous studies have also shown that least-cost modelling derived accumulated-cost values are better than straight-line distance at explaining ecological patterns that are affected by dispersal [43]–[46]. However, unlike previous studies, we have not only defined a cost-surface, but we have also taken an extra step by measuring actual dispersal events in terms of accumulated-cost to also define an accumulated-cost dispersal kernel. It is this novel combination of a cost-surface and accumulated-cost dispersal kernel that we can envisage could be used to improve dispersal models in three different ways.

If a dispersal event is modelled as a single discrete movement, the probability density function of a dispersal kernel could be used to describe the likelihood of the dispersal event having a specific accumulated-cost value. This could then be used in combination with a cost-surface to randomly select an end point for the dispersal event. This is essentially an equivalent process as that used by the straight-line distance dispersal approach, but with the benefit of recognising landscape structure.

It has also been suggested that a dispersing organism is increasingly likely to settle as accumulated transfer costs increase [1]. If the cost-surface is used as the basis for a random walk dispersal model, then the accumulated-cost could grow with each step in the random walk [47]. By using the cumulative distribution function of a dispersal kernel to convert the accumulated-cost into a cumulative distribution value, this approach could be used to assess the likelihood of settlement at each step of the random walk.

There are a variety of least-cost modelling approaches that aim to identify the area around a point that is likely to be reached [48]–[50]. Given the cost-surface and a dispersal limit, such as the maximum dispersal value or a cumulative distribution threshold, the area that could be reached can be delineated. The likelihood of reaching locations within this area could even be determined by converting the accumulated-cost values into probabilities through the use of an inverted cumulative distribution function.

With these different applications in mind, it is important to highlight that the landscape genetics approach we used to calibrate our cost-surface landscape representations is not the only or necessarily the best approach. While gene flow and dispersal are generally correlated they are not synonymous, as contemporary genetic structure is affected by other processes such as historical landscape change and population level survival and reproduction rates [31]. Therefore, other approaches to calibrating cost-surfaces that make use of detection, mark-recapture, or radio-telemetry data may provide a better option for some studies [6]. It is also worth noting that the LCPs between the start and end points of dispersal that were used to calibrate the accumulated-cost dispersal kernel are likely to underestimate the true transfer cost. The LCP finds the optimum route, which an animal is unlikely to actually take, and in some instances the start and end points are likely to represent truncated dispersal events, as dispersal may have continued beyond the period of observation. However, this use of an unrealistic shortest path is equivalent to the straight-line distance approach to estimating a distance dispersal kernel. This problem could be resolved by using a different approach to estimating the connectivity between the start and end points of a dispersal event such as directed random walks [47] or, more ideally, by using actual dispersal pathway data to produce an actual measure of the direct transfer costs. Dispersal pathway data would also improve the confidence in defining the start and end points of a dispersal event by enabling changes in behaviour to be identified [51].

It is also important to remember that we focussed on the direct costs associated with the transfer stage of dispersal, which is only one part of the broader whole dispersal process that also includes departure and settlement costs [1]. While this obviously means that models of dispersal must add departure and settlement costs to the transfer costs [2], it also means that our transfer costs need to be carefully interpreted as they may have been affected by costs incurred at other stages, as organisms will attempt to minimise the overall dispersal cost [1]. For example, lower departure costs resulting from better resources at the departure location may enable an organism to tolerate higher transfer costs, while higher settlement costs resulting from competitors at the settlement location may force an organism to minimise transfer costs. This means that when transfer costs are established without reference to the other departure and settlement costs, there is the potential for the transfer costs to misleading. Therefore, ideally dispersal costs should be calculated with reference to the whole dispersal process – for which our least-cost modelling approach could still be used to quantify the direct transfer costs. However, monitoring an organism through its entire dispersal process is of course a difficult task, so when this is not possible, we would suggest that direct transfer costs are established using observations of dispersal that, like our possum dispersal events, consisted of a large number of individuals from different locations and times in the hope of minimising any potential spatio-temporal biases.

To conclude, a new generation of dispersal models is required that better exploits recent empirical advances in understanding the dispersal process [2]. We believe that recent advances in the application of least-cost modelling in ecology provide an excellent basis for meeting this challenge. The novel combined cost-surface and accumulated-cost dispersal kernel approach that we have presented will hopefully enable more realistic modelling of the direct transfer costs associated with the dispersal process.
